# A Novel *MYRF* Variant Presenting With Scimitar Syndrome, Hepatopulmonary Fusion, Right‐Sided Congenital Diaphragmatic Hernia, and Uterine Didelphys

**DOI:** 10.1155/crpe/8646210

**Published:** 2026-08-17

**Authors:** Gul Sher, Samantha Weaver, Rahul Adwani, Jai Parkash Udassi

**Affiliations:** ^1^ Department of Pediatrics, Children’s Heart Center, WVU Golisano Children’s Hospital, Morgantown, West Virginia, USA; ^2^ WVU School of Medicine, Morgantown, West Virginia, USA

**Keywords:** cardiac-urogenital syndrome, hepatopulmonary fusion syndrome, *MYRF* mutation

## Abstract

The *MYRF* gene encodes a pleiotropic transcription factor essential for the development of multiple organ systems, including the heart, lungs, diaphragm, and genitourinary tract. Pathogenic variants in *MYRF* are associated with a multisystem disorder commonly referred to as MYRF‐related cardiac‐urogenital syndrome (CUGS). We describe a term female neonate with a maternally inherited likely pathogenic *MYRF* variant (c.1305_1311 + 1dup), a splice‐site duplication predicted to disrupt normal gene function. The patient presented with complex congenital anomalies, including scimitar syndrome, right‐sided congenital diaphragmatic hernia with hepatopulmonary fusion, pulmonary hypoplasia, and uterine didelphys. Several of these features have been individually reported in association with *MYRF*; however, uterine didelphys represents a previously unreported Müllerian duct anomaly within the MYRF‐related phenotypic spectrum. The clinical course was complicated by severe pulmonary hypertension, refractory hypoxemia, and necrotizing enterocolitis, culminating in neonatal death despite aggressive medical management. This case highlights a severe neonatal presentation of MYRF‐related disease in a female patient with an inherited pathogenic variant and expands the recognized phenotypic spectrum to include uterine didelphys. Recognition of sex‐specific manifestations and variable penetrance in MYRF‐related disorders is important for accurate diagnosis, prognostication, and genetic counseling in neonates with multisystem congenital anomalies.

## 1. Introduction

The myelin regulatory factor (*MYRF*) gene encodes a pleiotropic, membrane‐bound transcription factor that plays a critical role in human development [1]. Although initially characterized for its essential function in oligodendrocyte differentiation and central nervous system myelination, *MYRF* has since been recognized as an important regulator of multiple embryologic pathways, including cardiac, pulmonary, diaphragmatic and genitourinary development [2]. Pathogenic variants in *MYRF* are associated with a multisystem disorder commonly referred to as MYRF‐related cardiac‐urogenital syndrome, characterized by congenital heart defects, genitourinary anomalies, congenital diaphragmatic hernia, pulmonary hypoplasia and variable neurodevelopmental involvement [3, 4].

The phenotypic expression of MYRF‐related disorders is notably heterogeneous, with substantial variability in organ involvement, disease severity and clinical outcome [3]. Both de novo and inherited pathogenic *MYRF* variants have been reported, and incomplete penetrance with marked intrafamilial variability further complicates clinical recognition and diagnosis [5]. As additional cases are described, the clinical and genetic spectrum of MYRF‐related disease continues to expand, particularly with respect to sex‐specific manifestations and uncommon associated anomalies [6, 7].

Here, we report a term female neonate with a maternally inherited likely pathogenic *MYRF* variant presenting with scimitar syndrome, right‐sided congenital diaphragmatic hernia with hepatopulmonary fusion, pulmonary hypoplasia and uterine didelphys. While several of these features have been individually described in association with *MYRF*, uterine didelphys represents a previously unreported Müllerian duct anomaly within this disorder.

## 2. Case Presentation

Our patient was a term female neonate born at 39 weeks’ gestation via cesarean delivery, with Apgar scores of 3 and 7 at 1 and 5 min, respectively. She required intubation shortly after admission to the Pediatric Cardiac Intensive Care Unit for acute respiratory failure and was initiated on prostaglandin E1 infusion.

**FIGURE 1:**
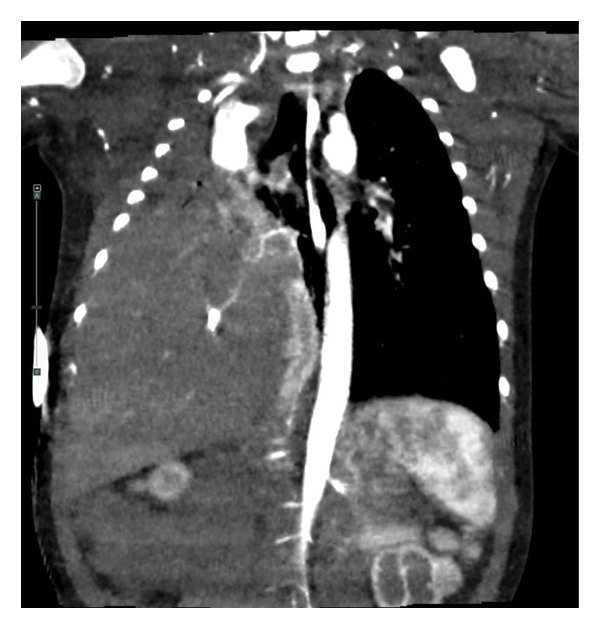
Right‐sided diaphragmatic hernia with hepatopulmonary fusion.

Prenatal diagnostic testing obtained by amniocentesis included fluorescence in situ hybridization (FISH), conventional karyotyping and chromosomal microarray analysis, all of which were normal. Prenatal echocardiography demonstrated a large, anteriorly malaligned ventricular septal defect with right‐to‐left shunting and a hypoplastic aortic arch. Postnatal cardiac evaluation confirmed these findings and further identified a severely hypoplastic right pulmonary artery (approximately 1 mm), a large patent ductus arteriosus with bidirectional shunting, moderate tricuspid regurgitation, hypoplasia of the distal aortic arch and partially anomalous pulmonary venous return, consistent with scimitar syndrome. Chest radiography showed opacification of the right hemithorax, raising concern for pulmonary hypoplasia. Subsequent cross‐sectional imaging with computed tomography and magnetic resonance imaging demonstrated a hypoplastic right lung, hepatopulmonary fusion, a right‐sided congenital diaphragmatic hernia with herniation of the liver into the thoracic cavity and subdiaphragmatic pulmonary sequestration (Figure 1). Abdominal ultrasound additionally demonstrated uterine didelphys. Physical examination demonstrated normal female external genitalia without documented genital ambiguity.

Rapid whole‐exome sequencing (GeneDx XomeDxXpress) identified a maternally inherited heterozygous *MYRF* (c.1305_1311 + 1dup) variant, classified by the testing laboratory as likely pathogenic. The variant affects a canonical splice donor site and is predicted to disrupt normal RNA splicing. A maternally inherited *RYR2* p.(E1742D) variant classified as a variant of uncertain significance was also identified. Mitochondrial genome sequencing and deletion analysis were negative. The *MYRF* variant provided a plausible candidate genetic explanation for the patient’s multisystem congenital anomalies, consistent with previously reported MYRF‐related phenotypes, including cardiac, pulmonary, diaphragmatic, and genitourinary involvement.

The patient’s clinical course was marked by severe hypoxemia, persistent hypercarbia, pulmonary hypertension, and poor lung compliance in the setting of right‐sided congenital diaphragmatic hernia and pulmonary hypoplasia. She required tracheostomy and gastrostomy tube placement to facilitate ventilation and feeding. Her pulmonary hypertension was treated with inhaled nitric oxide, sildenafil, and bosentan. Despite aggressive medical management, including treatment of recurrent pulmonary hypertensive crises, severe respiratory acidosis, and necrotizing enterocolitis, her condition progressively deteriorated. After extensive multidisciplinary discussions with the family, care was redirected toward comfort measures, and the patient died peacefully.

## 3. Discussion

The *MYRF* gene encodes a membrane‐bound transcription factor essential for the coordinated development of multiple organ systems, including the central nervous system, heart, lungs, diaphragm, and genitourinary tract [1, 4]. Pathogenic variants in *MYRF* have been associated with a spectrum of congenital anomalies, most prominently congenital heart defects, congenital diaphragmatic hernia, pulmonary hypoplasia, and genitourinary abnormalities—a constellation now commonly referred to as MYRF‐related cardiac‐urogenital syndrome [3, 4].

Since the initial description of MYRF‐related syndromic disease in 2018 [8], reported phenotypes have demonstrated substantial variability, including differences in organ involvement, severity, and survival. The majority of reported pathogenic *MYRF* variants are de novo and occur in male patients, although female patients and inherited variants with incomplete penetrance have increasingly been recognized [3, 4]. This growing body of literature underscores both the pleiotropic nature of *MYRF* and the challenges of genotype–phenotype correlation (Table 1).

**TABLE 1:** Comparison of clinical features in published MYRF‐related syndromes and the present case.

Feature	Pinz et al. 2018 [8]	Qi et al. 2018 [4]	Rossetti et al. 2019 [3]	Favier et al. 2024 [9]	Present case
Number of patients	2	7	3	12 (prenatal)	1
Sex	Male	Male‐predominant	Male	Both	Female
*MYRF* variant type	De novo	De novo	Mostly de novo	De novo/inherited	Inherited (maternal), likely pathogenic
Congenital heart disease	Yes	Yes	Yes (2/3^rd^))	Yes	Yes (scimitar syndrome, VSD, arch hypoplasia)
Scimitar syndrome	Yes	Yes (1/7^th^)	Yes (1/3^rd^)	Rare	Yes
Congenital diaphragmatic hernia	Yes	Yes	Yes	Yes	Yes (right‐sided)
Pulmonary hypoplasia	Yes	Yes	Common	Common	Yes
Hepatopulmonary fusion	Rare	Rare	Not specified	Not specified	Yes
Genitourinary anomalies	Yes	Yes	2/3^rd^	Yes	Yes
Absent uterus	Reported	Reported	Reported	Reported	No
Uterine didelphys	Not reported	Not reported	Not reported	Not reported	Yes
Survival	Variable	Variable	Variable	Variable	Neonatal death

Several of the anomalies observed in our patient have been individually described in association with *MYRF* pathogenic variants. Congenital heart defects and congenital diaphragmatic hernia have been well documented, while scimitar syndrome and hepatopulmonary fusion are relatively rare anomalies in patients with *MYRF* [3, 8].

The principal contribution of this report lies not in the identification of individual anomalies previously associated with *MYRF* but in the recognition of uterine didelphys as a Müllerian duct anomaly within the MYRF‐related phenotypic spectrum. While genitourinary abnormalities are common in MYRF‐related disease–most notably ambiguous genitalia, absent uterus, or disorders of sex development [6, 7]–uterine didelphys has not, to our knowledge, been previously reported in association with *MYRF* pathogenic variants. This distinction is important, as uterine didelphys represents a specific defect in Müllerian duct fusion rather than agenesis, suggesting a potentially broader role for *MYRF* in female reproductive tract development than previously appreciated.

In addition, this case highlights a severe neonatal presentation in a female patient, which remains underrepresented in the existing literature. Early reports of MYRF‐related cardiac‐urogenital syndrome predominantly described male patients, and although female cases have since been reported, detailed characterization of female reproductive tract anomalies remains limited. The presence of uterine didelphys in this patient further supports sex‐specific phenotypic variability and reinforces the need for systematic evaluation of internal reproductive anatomy in affected females (Table 1).

Another notable aspect of this case is the identification of a maternally inherited likely pathogenic *MYRF* variant. The mother had no known history of congenital heart disease, diaphragmatic defects, pulmonary disease, ophthalmologic abnormalities, or genitourinary anomalies. However, comprehensive evaluation for subtle manifestations, including dedicated cardiac imaging, ophthalmologic examination, and reproductive tract imaging, was not performed. Therefore, reduced penetrance, variable expressivity, or unrecognized subclinical manifestation cannot be excluded. This observation supports prior reports of marked intrafamilial variability among individuals with MYRF‐related disorders [5].

The identified *MYRF* variant (c.1305_1311 + 1dup) was classified by the clinical testing laboratory as likely pathogenic. The variant involves a duplication affecting a canonical splice donor site and is predicted to disrupt normal RNA splicing, potentially resulting in a loss of normal *MYRF* function [8, 10]. While the patient’s phenotype is highly consistent with MYRF‐related disease, functional RNA studies were not performed, and therefore, the precise molecular consequences of this variant remain inferred rather than experimentally confirmed.

Limitations of this report include the absence of functional RNA studies, the lack of a comprehensive phenotypic evaluation of the mother, which limits assessment of potential subclinical manifestations and genotype‐phenotype correlation, and the inherent constraints of a single‐case observation.

This case expands the phenotypic spectrum of MYRF‐related disease by identifying uterine didelphys as a previously unreported genitourinary manifestation and by illustrating the severity of disease that can occur in female patients with inherited pathogenic variants. Early recognition of MYRF‐associated presentations may facilitate more accurate prognostication, guide comprehensive multisystem evaluation—including detailed genitourinary imaging in females—and inform family counseling. Continued aggregation of well‐phenotyped cases will be essential to refine the understanding of MYRF‐related syndromes and their clinical variability.

## Author Contributions

All authors have contributed to the work, and reviewed and approved the content of the article. None of the article’s content is under consideration elsewhere or has been published in any other journal.

## Funding

The authors declare they have not received any financial support or funding for this article’s research, authorship, or publication.

## Consent

The authors confirm that consent was taken from the parent for the publication of this case report.

## Conflicts of Interest

The authors declare no conflicts of interest.

## Supporting Information

Additional supporting information can be found online in the Supporting Information section.

## Supporting information


**Supporting Information** CARE checklist of information to include when writing a case report.
